# Neovaginal Cytologic and Molecular Screening Findings for Human Papillomavirus and Sexually Transmitted Infections Among Transgender Women

**DOI:** 10.1093/ofid/ofag516

**Published:** 2026-08-14

**Authors:** Teresita de Jesús Cabrera López, Marla Naivi Toiber Rodríguez, Elizabeth Vancini Nieto, Mitzi Zaira Fong Ponce, Esmeralda Román Mar, Rebeca Eunice García Mendiola, Juan Manuel Bello López

**Affiliations:** Obstetrics and Gynecology, Colposcopy, HIV, Clínica Especializada Condesa Iztapalapa, Ciudad de México, México; Psychology, Clínica Especializada Condesa Iztapalapa, Ciudad de México, México; Sexology, Clínica Trans, Clínica Especializada Condesa Iztapalapa, Ciudad de México, México; Endocrinology, Clínica Trans, Clínica Especializada Condesa Cuauhtémoc, Ciudad de México, México; Endocrinology, Clínica Trans, Clínica Especializada Condesa Cuauhtémoc, Ciudad de México, México; Clinical Chemistry, Medical Laboratory, Clínica Especializada Condesa Iztapalapa, Ciudad de México, México; Clinical Chemistry, Medical Laboratory, Hospital Juárez de México, Ciudad de México, México

**Keywords:** human papillomavirus (HPV), molecular screening, neovagina, sexually transmitted infections (STI), transgender women

## Abstract

Evidence regarding optimal screening strategies for neovaginal health in transgender women remains limited. Data describing cytologic and molecular detection of human papillomavirus (HPV) and sexually transmitted infections (STIs) in neovaginal tissue are scarce, particularly in populations with high burden of HIV and STI. We conducted a descriptive observational study of transgender women with neovagina receiving care at a specialized sexual health clinic in Mexico City. Neovaginal samples were collected for cytologic evaluation and polymersase chain reaction (PCR)-based molecular testing for HPV and sexually transmitted pathogens. Sixteen transgender women were included (median age 44.5 years; range 31-52 years). Cytology samples were obtained in 13 participants and were negative in all cases. Despite universally negaive cytology, PCR-based molecular testing identified high-risk HPV in 38.5% of tested participants and detected other sexually transmitted pathogens *(Chlamydia trachomatis, Mycoplasma hominis, Neisseria gonorrhoeae, and Ureaplasma urealyticum)*. Molecular screening identified HPV infection and other sexually trnsmitted pathogens in neovaginal samples despite negative cytology. These findings highlight potential limitations of cytology-based screening and support the integration of molecular diagnostics as part of comprehensive sexual health care for transgener women.

## Graphical Abstract

**Graphical Abstract:**
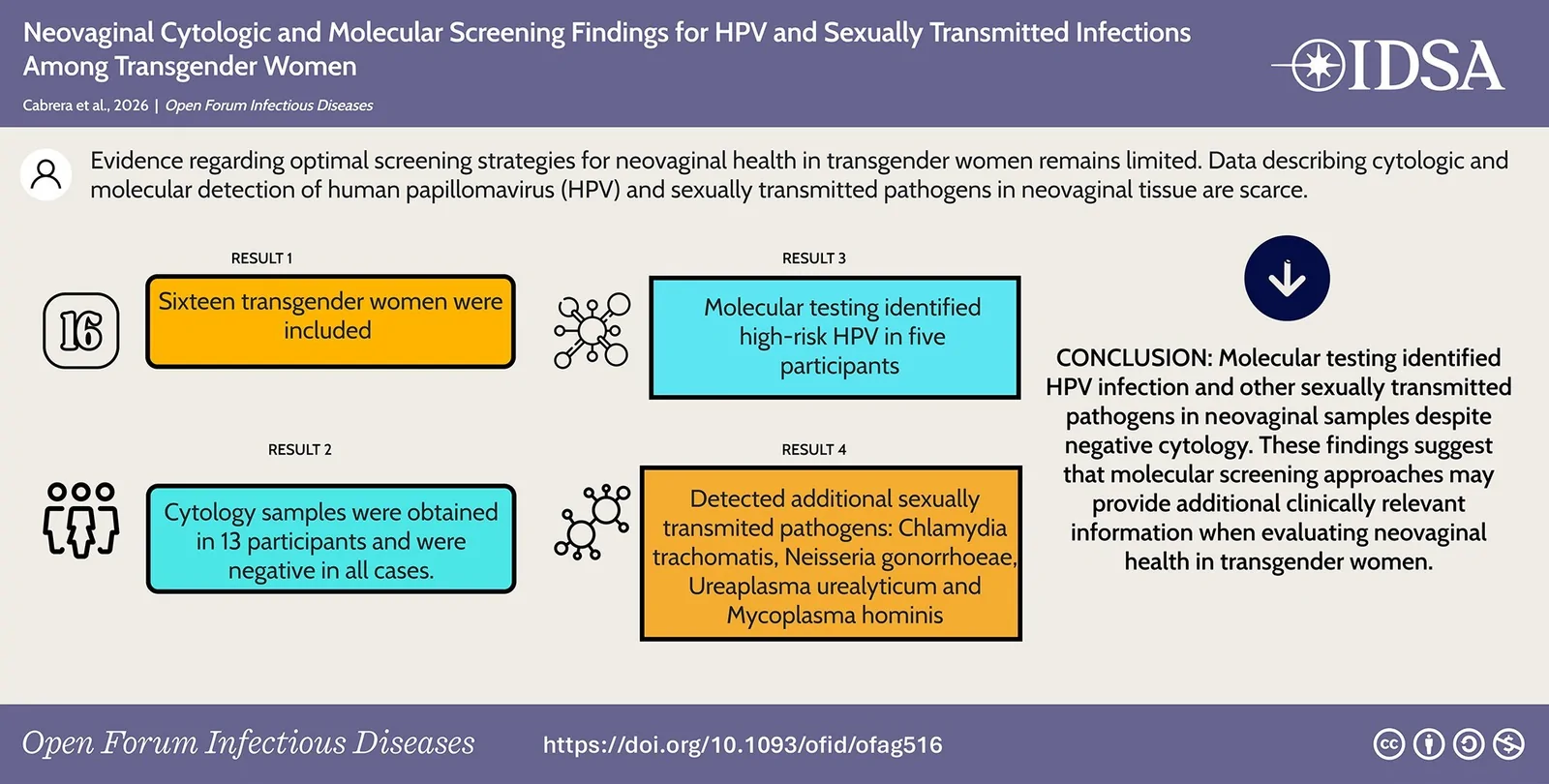
This graphical abstract is also available at Tidbit: https://tidbitapp.io/tidbits/molecular-detection-of-high-risk-hpv-and-sexually-transmitted-pathogens-in-neovaginal-samples-from-transgender-women-in-mexico-city?utm_campaign=tidbitlinkshare&utm_source=IO.

## BACKGROUND

As increasing numbers of transgender women undergo gender-affirming vaginoplasty worldwide, clinicians are increasingly confronted with questions regarding long-term neovaginal cancer prevention and sexually transmitted infections (STI) screening. However, evidence-based recommendations remain almost nonexistent. Neovaginal reconstruction most commonly involves penile and scrotal skin inversion vaginoplasty, although other techniques using intestinal segments or alternative tissues may also be employed. As access to gender-affirming surgery has increased globally, clinicians are encountering a larger number of individuals with neovaginal anatomy who require ongoing gynecologic and infectious disease care. However, clinical evidence regarding the long-term gynecologic and infectious outcomes of neovaginal tissue remains limited [1, 2].

Human papillomavirus (HPV) infection is the most common sexually transmitted viral infection worldwide and is strongly associated with the development of anogenital dysplasia and cancer [3]. Transgender women may be at increased risk of HPV infection due to ongoing sexual exposure and a high burden of HIV and STI in this population [4]. Previous studies have demonstrated the presence of high-risk HPV in neovaginal tissue following vaginoplasty, even in the absence of cytologic abnormalities, supporting the potential role of molecular testing in this setting [5]. Human papillomavirus infection in transgender women with neovaginas has emerged as an important area of interest because persistent infection with oncogenic HPV types may lead to benign and malignant neovaginal lesions. However, unlike cervical HPV infection in cisgender women, the epidemiology, natural history, and optimal screening strategies for neovaginal HPV infection remain poorly defined. A recent systematic review and meta-analysis including 21 studies estimated an overall genital HPV prevalence of ∼15% among transgender women with neovaginas and identified HPV 16 as the most frequently detected genotype. Despite these findings, available evidence remains limited by small sample sizes, heterogeneous sampling methods, and the absence of longitudinal studies, precluding evidence-based recommendations for routine neovaginal screening [6].

In addition to HPV, transgender women with neovaginas may also develop other STIs. Current guidelines recommend that STI screening in transgender persons be tailored to individual anatomy, sexual practices, and sites of exposure; however, evidence specific to neovaginal screening remains limited [7].

Cytologic evaluation of neovaginal samples has been described in small series, but interpretation may be challenging due to histologic differences between neovaginal and cervical epithelium [5]. Although HPV-related neovaginal disease appears to be relatively common, reports of HPV associated neovaginal squamous cell carcinoma remain rare. However, increasing access to gender-affirming surgery may lead to a growing number of transgender women with long-term neovaginal exposure to HPV-related disease. Recent reports highlight that optimal screening strategies for dysplasia and malignancy in neovaginal tissue remain undefined, and no formal recommendations currently exist regarding surveillance, follow-up, or management of abnormal findings [8, 9].

In this study, we describe cytologic and molecular screening findings for HPV and other sexually transmitted pathogens among transgender women with neovagina receiving care at a specialized clinic in Mexico City.

## METHODS

### Study Design and Setting

We conducted a cross-sectional observational study of transgender women with neovagina receiving care at Clínica Especializada Condesa Iztapalapa, a specialized public HIV and sexual health clinic in Mexico City, Mexico. This clinic provides comprehensive care for people with HIV and sexual health services for key populations, including transgender persons.

Participants were identified during routine clinical care between March 2025 and December 2025. Participants were recruited consecutively through active referral from healthcare personnel at both participating clinics and through community outreach using digital media. Participation was voluntary, and no financial incentives were provided.

### Study Population

Transgender women with a history of gender-affirming vaginoplasty and a constructed neovagina who attended the clinic during the study period were eligible for inclusion. All participants had undergone gender-affirming vaginoplasty using penile and scrotal skin inversion techniques. A total of 16 transgender women were included in the study. The median age was 44.5 years (range 31–52 years). Three participants living with HIV were receiving care at the same clinic.

### Sample Collection

Neovaginal samples were collected under direct visualization using a rhinoscope, anoscope, or small vaginal speculum depending on neovaginal dimensions. Cytology samples were collected using a cytobrush and Ayre spatula, with sampling directed to the neovaginal apex/fundus, successfully obtained in 13 participants. In 3 cases, samples could not be collected: 2 due to neovaginal closure and 1 because the patient declined sample collection. Samples were used for cytologic evaluation and molecular testing.

### Cytologic Evaluation

Neovaginal cytology specimens were processed and interpreted according to standard cytologic evaluation procedures used for lower genital tract cytology.

### Molecular Testing

Polymerase chain reaction (PCR)–based molecular testing was performed using PCR (Anyplex™ II STI-7e, Seegene) for detection of STIs, including *Chlamydia trachomatis, Neisseria gonorrhoeae, Mycoplasma genitalium, Mycoplasma hominis, Trichomonas vaginalis, Ureaplasma parvum*, and *Ureaplasma urealyticum*.

HPV PCR testing (Allplex™ HPV28, Seegene) was used for the detection of 9 low-risk HPV (6, 11, 40, 42, 43, 44, 54, 61, and 70) and 19 high-risk genotypes (16, 18, 26, 31, 33, 35, 39, 45, 51, 52, 53, 56, 58, 59, 66, 68, 69, 73, and 82).

### Statistical Analysis

Descriptive statistics were used to summarize findings. Continuous variables were reported as medians and ranges, and categorical variables were summarized using counts and proportions.

## RESULTS

A total of 16 transgender women with neovagina were included in the study. Participants had undergone gender-affirming surgery at a median age of 38 years and had initiated gender-affirming hormone therapy at a median age of 20 years. The sociodemographic and clinical characteristics of participants are summarized in Table 1. Characteristics stratified by HIV status are summarized in Table 2. Cytologic and molecular findings are summarized in Table 3.

**Table 1.** Sociodemographic and Clinical Characteristics of Participants (n = 16)

Characteristic	Value	
Total participants	16	
Median age, y (range)	44.5 (31–52)	
With HIV, n (%)	3 (18.7)	
Education, n (%)	Secondary school	4 (25)
	High school	7 (43.8)
	University	5 (31.3)
Employment, n (%)	Paid employment	14 (87.5)
	Homemaker	1 (6.3)
	Student	1 (6.3)
Current relationship status, n (%)	Has a partner	9 (56.3)
	Single	7 (43.8)
Age at gender-affirming surgery, y, median (range)	38 (25–49)	
Age at initiation of gender-affirming hormone therapy, y, median (range)	20 (15–31)	
Time from initiation of gender-affirming hormone therapy to gender-affirming surgery, y, median (range)	18 (2–27)	
Cytology and PCR		
Cytology obtained	13 (81.3%)	
Cytology not obtained	3 (18.7%) Neovaginal closure 2, patient declined sampling 1	
HPV PCR performed	13 (81.3%)	
High-risk (HR) HPV infection detected	5 (38.5% of those tested) (HR HPV:16, 31, 35, 39, 59, 66, 68)	
Other pathogens detected	*Chlamydia trachomatis*, *Mycoplasma hominis*, *Neisseria gonorrhoeae*, *Ureaplasma urealyticum*	

Abbreviations: HPV, human papillomavirus; PCR, polymerase chain reaction.

**Table 2.** Characteristics Stratified by HIV Status

Characteristic	Total (n = 16)	With HIV (n = 3)	Without HIV (n = 13)
Age, median (range), y	44.5 (31–52)	41 (40–50)	45 (31–52)
Current sexual activity, n (%)	10 (62.5)	1 (33.3)	9 (69.2)
Current relationship status, n (%)	9 (56.3)	0	9 (69.2)
Cytology performed, n (%)	13 (81.3)	1 (33.3)	12 (92.3)
HPV PCR performed, n (%)	13 (81.3)	2 (66.7)	11 (84.6)
High-risk HPV infection detected, n (%)	5 (38.5)	0	5 (38.5)
Other STI detected, n (%)	4 (25.0)	2 (66.7)	2 (15.4)

Abbreviations: HPV, human papillomavirus; PCR, polymerase chain reaction; STI, sexually transmitted infection.

**Table 3.** Cytologic and Molecular Findings

Patient	Cytology	HPV PCR (Category)	HPV HR Genotypes	HPV LR Genotypes	STI PCR Panel	With HIV
P001	Negative	Negative	…	…	*Ureaplasma urealyticum*	Yes^a^
P002	Negative	Positive HPV HR + LR	66, 35	54, 61, 6, 40	Negative	No
P003	Negative	Negative	…	…	Inadequate	No
P004	Negative	Inadequate	…	…	Inadequate	No
P005	Negative	Negative	…	…	Negative	No
P006	Negative	Negative	…	…	Negative	No
P007	Negative	Negative	…	…	Negative	No
P008	NP					No
P009	Negative	Negative	…	…	Negative	No
P010	Negative	Positive LR	…	42, 43	*Chlamydia trachomatis*, *Neisseria gonorrhoeae*, *U. urealyticum*	No
P011	NP					Yes ^a^
P012	Negative	Positive HR	16	…	Negative	…
P013	Negative	Positive HR	16, 31	…	Negative	No
P014	NP	Negative	…	…	*Mycoplasma hominis*, *U. urealyticum*	Yes ^a^
P015	Negative	Positive HR + LR	66, 59, 39, 68	70, 61, 44	*M. hominis*, *N. gonorrhoeae*	No
P016	Negative	Positive HR + LR	31	61	Negative	No

Abbreviations: HPV, human papillomavirus; NP, not performed; PCR, polymerase chain reaction; HR, high-risk; LR, low-risk; STI, sexually transmitted infection.

^a^All participants with HIV had viral suppression (<40 copies/mL); CD4 counts were 1001, 504, and 459 cells/mm^3^.

Polymerase chain reaction–based molecular testing identified high-risk HPV infection in 5 participants. Notably, all available neovaginal cytology specimens were negative despite detection of high-risk HPV in 38.5% of participants undergoing PCR-based molecular testing. High-risk HPV genotypes detected included HPV 16, 31, 35, 39, 59, 66, and 68.

Polymerase chain reaction testing also detected additional sexually transmitted pathogens including *C. trachomatis*, *N. gonorrhoeae*, *U. urealyticum*, and *M. hominis*.

## DISCUSSION

In our cohort, all participants had undergone vaginoplasty using penile and scrotal skin inversion techniques, resulting in neovaginal epithelium composed primarily of keratinized squamous tissue derived from genital skin rather than cervical mucosa. Despite universally negative cytology findings, PCR-based molecular testing identified high-risk HPV infection, suggesting that molecular screening may provide clinically relevant information beyond cytology alone in this population.

Previous studies have documented HPV infection in neovaginal tissue following gender-affirming vaginoplasty, with reported prevalence of high-risk HPV ranging from approximately 8%–20% [5, 6]. Consistent with these findings, HPV infection in our cohort was identified despite negative cytologic results, highlighting the potential limitations of cytology-based screening in this setting.

Interpretation of neovaginal cytology presents unique challenges due to the histologic characteristics of neovaginal epithelium, which differs from cervical epithelium [5]. As a result, cytologic evaluation alone may underestimate HPV infection, supporting the integration of molecular testing strategies for more accurate detection.

Evidence from other noncervical screening settings further supports these limitations. For example, anal cytology screening has demonstrated important diagnostic challenges, including high rates of inadequate samples and suboptimal sensitivity for detecting clinically significant lesions. These findings suggest that cytology alone may underestimate HPV-related disease in noncervical squamous epithelium. Consistent with this concern, all neovaginal cytology samples in our cohort were negative despite molecular detection of high-risk HPV in multiple participants, supporting the complementary role of molecular HPV testing in neovaginal screening strategies [10].

In addition to HPV, PCR-based molecular testing also identified other sexually transmitted pathogens, including *C. trachomatis*, *N. gonorrhoeae*, *U. urealyticum*, and *M. hominis*. These findings underscore the importance of comprehensive sexually transmitted infection screening in transgender women receiving sexual health care, particularly in populations with a high burden of HIV and other STIs [4, 11]. Current recommendations emphasize that STI screening in transgender persons should be individualized according to sexual practices, anatomy, and exposure sites rather than gender identity alone [7].

Recent case reports of HPV-related neovaginal squamous cell carcinoma further emphasize the importance of improving screening strategies in transgender women with neovaginal reconstruction. Although malignant transformation appears to be rare, the true incidence remains unknown and may increase as access to gender-affirming surgery expands. Importantly, there are currently no evidence-based recommendations regarding screening intervals, preferred diagnostic methods, or management of abnormal neovaginal findings. Additionally, whether cytologic screening in neovaginal tissue performs comparably to cervical cytology remains unclear, suggesting that molecular HPV testing may play an important complementary role in future screening strategies [9].

Importantly, there are currently no standardized guidelines for screening neovaginal tissue for HPV or other STIs. Existing international guidelines for HPV prevention and sexually transmitted infection management do not specifically address neovaginal anatomy in transgender women, highlighting a critical gap in clinical guidance [12–14]. This gap is particularly relevant given the disproportionate burden of HIV and STIs in transgender women and the limited inclusion of this population in screening research [11, 15, 16]. Additionally, existing evidence suggests that HPV-related disease may present differently in noncervical epithelial tissues, further complicating screening strategies in neovaginal anatomy [17].

Our findings support the potential role of integrating molecular screening into routine care to improve detection of infections that may remain unrecognized using cytology alone. From a public health perspective, expanding screening strategies to include molecular diagnostics may contribute to earlier detection and improved sexual health outcomes in this population.

Our findings are consistent with the recent systematic review and meta-analysis by Elasifer et al, which reported an overall genital HPV prevalence of 15% among transgender women with neovaginas and identified HPV 16 as the most commonly detected genotype. Similar to previous reports, high-quality evidence remains scarce because published studies include relatively small cohorts and considerable methodological heterogeneity regarding neovaginal sampling techniques, HPV assays, and specimen adequacy. These limitations currently preclude the development of standardized screening recommendations and highlight the need for prospective studies using standardized sampling protocols and longitudinal follow-up.

HIV infection may represent an important modifier of neovaginal HPV persistence and progression risk. Studies have shown that people with HIV may exhibit increased prevalence of high-risk HPV genotypes, higher rates of multiple HPV infections, and greater HPV-related disease burden, likely related to impaired immune control of HPV replication [18, 19]. Reduced HPV clearance in this population may contribute to persistent infection and increased risk of HPV-related dysplasia and malignant transformation, which may have important implications for neovaginal screening strategies. In our cohort, no high-risk HPV infection was identified among participants with HIV; however, interpretation is limited by the small number of participants, incomplete testing, and the fact that all individuals with HIV had virologic suppression with relatively preserved immune status.

This study has several limitations. The sample size was small and reflects a single-center experience. Additionally, cytology samples could not be obtained in all participants due to anatomical factors or patient preference. Prospective longitudinal cohort studies are needed to determine HPV persistence, viral clearance, the predictive value of cytology and molecular testing, and optimal screening intervals for transgender women with neovaginas.

Additionally, anal cytology and anal HPV testing were not systematically performed, limiting our ability to assess concordance of HPV infection across anatomical sites. Future prospective longitudinal studies incorporating multisite HPV testing may better define HPV persistence, anatomical concordance, viral clearance, and site-specific disease progression.
